# Acquired Hemophilia A Successfully Treated with Rituximab

**DOI:** 10.4084/MJHID.2015.024

**Published:** 2015-03-01

**Authors:** Giovanni D’Arena, Elvira Grandone, Matteo Nicola Dario Di Minno, Pellegrino Musto, Giovanni Di Minno

**Affiliations:** 1 Hematology and Stem Cell Transplantation Unit, IRCCS Centro di Riferimento Oncologico della Basilicata, Rionero in Vulture, Italy; 2 Hemostasis and Thrombosis Unit, IRCCS “Casa Sollievo della Sofferenza” Hospital, San Giovanni Rotondo, Italy; 3 Department of Clinical Medicine and Surgery, Regional reference Center for Coagulation Disorders, Federico II University, Naples, Italy; 4 Unit of cell and molecular biology in cardiovascular diseases, Centro Cardiologico Monzino, IRCCS, Milan, Italy; 5 Scientific Direction, IRCCS Centro di Riferimento Oncologico della Basilicata, Rionero in Vulture, Italy

## Abstract

Acquired hemophilia A (AHA) is a rare bleeding disorder due to the development of specific autoantibodies against factor VIII. The anti-CD20 monoclonal antibody Rituximab has been proven to be effective in obtaining a long-term suppression of inhibitors of AHA, besides other immunosuppressive standard treatments. Here we describe a case of idiopathic AHA in a 60-year old man successfully treated with rituximab. He showed a complete clinical response with a normalization of clotting parameters after 5 weekly courses of rituximab given at a dose of 375 mg/sqm., but after stopping rituximab, an initial worsening of coagulation parameters induced the addition of 3 further courses. At present, the patient is in complete clinical and hematological remission after 200 days. This case confirms that Rituximab may be a safe and useful tool to treat AHA and, a prolonged administration can overcome the initial resistance. However, the precise position of this drug in the therapeutic strategy (first or second-line, alone or in combination with other drugs) remains to be established and warrants further investigation.

## Introduction

Rituximab is a chimeric human/murine monoclonal antibody targeting CD20 antigen on B-cell surface.^1^ It is extensively used to treat CD20 positive hematologic malignancies and is now increasingly employed to treat several autoimmune disorders. Acquired hemophilia A (AHA), is a rare bleeding disorder caused by the development of specific autoantibodies, the so-called inhibitors, against naturally occurring factor VIII (FVIII), and has been treated with rituximab too.^2^,^3^

The treatment of such a disorder is aimed to control bleeding and to suppress inhibitors, as well.^4^,^5^ These results are usually obtained by using standard immunosuppressive therapy (steroids, cyclophosphamide, azathioprine). Rituximab is generally considered a second-line treatment option. About 171 patients have been treated with this particular approach so far.

Here we describe a paradigmatic patient with AHA, who experienced a complete and long-lasting hematological response to Rituximab.

## Case Report

A non-hemophilic 60-year old man was admitted to another Hospital because of the sudden appearance of subcutaneous hemorrhage in his upper right arm following minor trauma. Laboratory investigations revealed a markedly prolonged activated partial thromboplastin time (aPTT, 99 seconds,) (ratio 2.7), not corrected with normal plasma (1:1) after a 2 hour incubation: (Prothrombin time (PT) was in normal range). At the same time, heparin contamination, Lupus anticoagulants, and other autoimmune diseases were excluded. Hemoglobin level was 11.2 g/dL while platelets count was normal (252.000/μL). No other biochemical or clotting system abnormalities were identified. Antibodies against hepatitis B and C viruses and HIV were found negative. Neoplastic biomarkers and a total body tomography computerized scan were normal.

The clinical history was also negative for the use of drugs known to be associated with the development of AHA.

Consistent with a diagnosis of AHA, the coagulation factor assay revealed that FVIII levels were 2.6 %, with a titer of 4 Bethesda Units (BU), thus confirming the presence of an acquired FVIII inhibitor.

**Figure 1:**
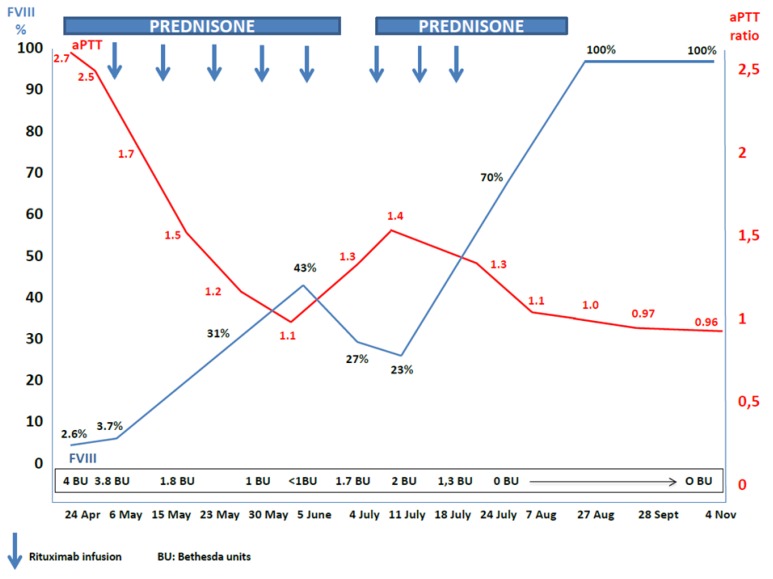
Schematic representation of the lab parameters course of the patient treated with rituximab for his AHA.

The patient initially received prednisone at a dose of 1 mg/Kg body weight orally given and, due to acute bleeding stage, a treatment with rFVIIa (Novoseven) was also started at a dose of 90 mcg/Kg every 2 hours (4 doses) and then every 4 hours for 6 doses. Despite the low inhibitor title at diagnosis, no response to corticosteroids was obtained. The bleeding persisted notwithstanding the prolonged treatment with rFVIIa. The patient was then sent to our Institution, where he initiated a treatment with Rituximab at a dose of 375 mg/sqm weekly in combination with prednisone. After the fifth dose, BU was undetectable and aPTT normalized and bleding stopped. For these reasons, prednisone was then slowly tapered, but at +57 days from the start of Rituximab therapy aPTT was found again prolonged (40 s; ratio 1.4) and FVIII levels reduced (27%) with 1.7 BU, in absence of any new hemorrhagic manifestation. Prednisone was then reintroduced at the dose of 1 mg/Kg and Rituximab given for 3 additional infusions, with normalization of aPTT and disappearance of inhibitor since a week after the eighth Rituximab infusion (Figure 1). The patient stopped prednisone therapy at +150 days form start of Rituximab without clinical signs of bleeding and normal clotting tests. At the last follow-up (+200 days) the patients is still in clinical and laboratory continue complete remission. Overall, Rituximab infusions were well tolerated, without evidence of infusion and/or late reactions. Finally, no infections have been reported so far.

## Discussion

AHA is a rare bleeding disorder due to acquired autoantibodies against FVIII. Its incidence has been estimated to be 0.2–1.0 case per million population per year, but it is probably underestimated.^3^ Soft tissue bleeding manifestations are often severe and may occur spontaneously or after minor trauma. Inhibitors are idiopathic in nature in approximately half of patients. In the remaining cases, various conditions are associated with FVIII inhibitors development, such as connective tissue and inflammatory bowel diseases, puerperium, malignancies, and dermatologic disorders.

An unexplained prolongation of the aPTT, not corrected by the in vitro addition of normal plasma (mixing test), is the typical laboratory feature of AHA. FVIII level is reduced, and the presence of an inhibitor is revealed by Bethesda assay. Treatment is aimed at the control of the acute bleeding and the suppression of the inhibitor, as well. Acute bleeding is managed through normalization of factor VIII level. Despite some authors recommend choosing hemostatic treatment, such as recombinant activated FVII (rFVII, Novoseven; Novo, Nordisk) and the activated prothrombin complex concentrate (APCC, FEIBA; Baxter Healthcare), according to the Bethesda assay (<5 BU or >5 BU, respectively), some considerations need. As reviewed in detail by Tiede et al, in contrast to congenital hemophilia, inhibitors in acquired hemophilia do not follow a log-linear dose-response relationship, that is the basis for quantification in Bethesda assay.^6^ In addition, an analysis of data from the EACH2 registry demonstrated that bypassing agents are more effective than FVIII infusion.^5^ Human FVIII, porcine FVIII, and desmopressin are also used. Immunosuppression is the mainstay to obtain inhibitor eradication. Steroids alone or combined with cytotoxic agents (i.e., cyclophosphamide, azathioprine, vincristine, or combination therapy), are also frequently used. The combined treatment of steroids with cyclophosphamide is able to eradicate inhibitors in about 70% of patients with AHA.

The monoclonal antibody Rituximab, is a chimeric antibody targeting CD20 antigen on B-cell surface.^1^ It has been reported to be effective in eradicating the inhibitors in AHA and 172 patients with this condition have been treated so far, including the present case. Rituximab has been used alone or in combination with other immunosuppressive drugs, such as steroids and cyclophosphamide, as salvage or first line-therapy. Overall, 157 patients (91%) showed a response, with 146 patients (85%) achieving complete response and 20 patients (12%) partial response. Five patients (3%) did not respond to rituximab therapy. The dose of Rituximab infused in almost all cases was 375 mg/sqm, similar to that employed in the lymphoma treatment. However, lower dose has been also used (100 mg). The number of rituximab infusions was very variable (from a single low-dose to 12 standard doses). In addition, in the majority of cases 4 standard doses were the schedule used and the time to response was also heterogeneous (from 1 week to more than one year).^7^ Of note, re-treatment with Rituximab was generally effective. Overall, the administration of Rituximab in AHA was well tolerated with very few infusional side effects, and no infectious complications have been reported, so far. We choose to treat our patient with eight weekly doses of rituximab according to the response to treatment (Figure 1). The present case suggests that in certain circumstances a more extensive course of rituximab infusions needs to reach a threshold of circulating B-lymphocytes whose antibodies production is not able to support the autoimmune disorder. In our patient, a B-cell number of 0.5/μL was achieved after the 8^th^ doses, when a complete response was also seen (Table 1).

At variance of cases reported by Franchini,^7^ the rate of a stable remission is much lower in some published reports: 71% by the European Acquired Haemophilia (EACH2) registry and 48% by the AHA Working group of the German, Austrian and Swiss Thrombosis and Hemostasis society (GTH).^5^,^8^ Finally, a treatment algorithm was proposed by Franchini and Mannucci very recently.^9^ Authors stated that first-line therapy for patients with AHA should be the association of prednisone and cyclophosphamide while Rituximab ± immunosuppressive therapy should be reserved for second-line treatment.

In conclusion, the use of anti-CD20 monoclonal antibody Rituximab is increasingly used to treat autoimmune disorders. In the case of AHA, its definitive role in eradicating inhibitors (first or second-line, high or low inhibitor titers, older and/or frail patients for whom corticosteroid and cytotoxic therapy are unsuitable) requires further evaluation. However, randomized-controlled trials are very hard to be designed because of the rarity of AHA. Notwithstanding additional data have to be acquired in order to optimize the use and to compare the efficacy of this drug to that of other treatments, current evidences confirm that Rituximab should be considered as part of the therapeutic armamentarium of AHA.

## Figures and Tables

**Table 1:** Circulating lymphocytes and B-cell number before and after Rituximab infusions

No. Rituximab infusion	Basal value	1^th^	2^th^	3^th^	4^th^	5^th^	6^th^	7^th^	8^th^
Lymphocytes/μL	2.600	2.000	1.100	1.200	1.100	1.000	1.000	1.100	1.200
B-cells/μL	564	320	190	89	70	1	0.7	0.5	0.5

Lymphocytes and B-cells (CD20+ lymphocytes evaluated by means of flow cytometry) were detected before the first (basal value) and after each rituximab infusion.
